# MAL promoter hypermethylation as a novel prognostic marker in gastric cancer

**DOI:** 10.1038/sj.bjc.6604777

**Published:** 2008-11-11

**Authors:** T E Buffart, R M Overmeer, R D M Steenbergen, M Tijssen, N C T van Grieken, P J F Snijders, H I Grabsch, C J H van de Velde, B Carvalho, G A Meijer

**Affiliations:** 1 Department of Pathology, VU University Medical Center, Amsterdam, The Netherlands; 2 Pathology and tumour biology, Leeds Institute of Molecular Medicine, University of Leeds, Leeds, UK; 3 Department of Surgery, Leiden University Medical Center, Leiden, The Netherlands

**Keywords:** gastric cancer, promoter hypermethylation, prognostic marker, MAL

## Abstract

T-lymphocyte maturation associated protein, MAL, has been described as a tumour-suppressor gene with diagnostic value in colorectal and oesophageal cancers, and can be inactivated by promoter hypermethylation. The aim of this study was to analyse the prevalence of *MAL* promoter hypermethylation and the association with mRNA expression in gastric cancers and to correlate methylation status to clinicopathological data. Bisulphite-treated DNA isolated from formalin-fixed and paraffin-embedded samples of 202 gastric adenocarcinomas and 22 normal gastric mucosae was subjected to real-time methylation-specific PCR (Q-MSP). Two regions within the *MAL* promoter (M1 and M2) were analysed. In addition, 17 frozen gastric carcinomas and two gastric cancer cell lines were analysed both by Q-MSP and real-time RT–PCR. Methylation of M1 and M2 occurred in 71 and 80% of the gastric cancers, respectively, but not in normal gastric mucosa tissue. Hypermethylation of M2, but not M1, correlated with significantly better disease-free survival in a univariate (*P*=0.03) and multivariate analysis (*P*=0.03) and with downregulation of expression (*P*=0.01). These results indicate that *MAL* has a putative tumour-suppressor gene function in gastric cancer, and detection of promoter hypermethylation may be useful as a prognostic marker.

Despite the overall decreasing rates of incidence and mortality, gastric cancer remains the second most common cause of cancer death worldwide (Parkin *et al*, 2005). The only possible curative treatment is surgery, but clinical outcome largely depends on the stage of the disease. Early detection of gastric cancer, before the tumour has metastasised to the lymph nodes, can therefore contribute to reducing deaths from gastric cancer. However, the knowledge on the molecular pathogenesis of gastric cancer and availability of possible biomarkers with clinical value is limited. Further insight in the molecular pathogenesis of gastric cancer will aid the discovery of new markers with high clinical relevance in gastric cancer, which are essential for improving gastric cancer prognosis.

Gastric cancers, like many other solid tumours, are characterised by the presence of genetic instability leading to the disruption of many genes, either resulting in their activation (oncogenes) or inactivation (tumour-suppressor genes). One of the common mechanisms of inactivation of tumour-suppressor genes is promoter hypermethylation (Baylin *et al*, 1998). Gene silencing by promoter hypermethylation has been described in gastric cancer for multiple genes, including *hMLH1*, involved in DNA mismatch repair, *CDH1*, involved in cell adhesion and the cell cycle regulator *p16* (Fleisher *et al*, 1999; Esteller *et al*, 2001; Machado *et al*, 2001; Carvalho *et al*, 2003). In addition, promoter hypermethylation of *Cox2* has been shown to be an independent prognostic marker in gastric cancer (de Maat *et al*, 2007).

In other gastrointestinal cancers, that is, colorectal and oesophageal cancer, the T-lymphocyte maturation associated protein MAL, involved in glycolipid-enriched membrane-mediated apical transport, has been described to be inactivated by promoter hypermethylation (Puertollano and Alonso, 1999; Mimori *et al*, 2003; Kazemi-Noureini *et al*, 2004; Mori *et al*, 2006; Lind *et al*, 2007). Promoter hypermethylation of *MAL* was a frequent event in these two cancer types, but infrequent in normal mucosa. As promoter hypermethylation of *MAL* could already be detected in cancer precursor lesions, it has been suggested as a tumour-suppressor gene with diagnostic value (Lind *et al*, 2007; Mimori *et al*, 2007). To the best of our knowledge, *MAL* promoter hypermethylation has not yet been shown in gastric cancers. Aim of this study was therefore to analyse promoter hypermethylation of *MAL* in gastric cancers, its relation to gene silencing and to determine its clinical value as a prognostic marker.

## Materials and methods

### Material

Two hundred and two formalin-fixed and paraffin-embedded (FFPE) gastric adenocarcinoma samples, randomly selected from the Leeds University archive and 22 normal gastric biopsy specimens, randomly selected from the archives of the VU University Medical Center, were included in this study. In addition, 17 snap-frozen gastric cancer tissue samples, obtained from the archives of the department of pathology of the VU University Medical Center (Weiss *et al*, 2003) were included. Patients did not receive chemotherapy, nor radiotherapy. Moreover, two gastric cancer cell lines, IPA220 and GP202 (Gartner *et al*, 1996), kindly provided by Professor Dr R Seruca (IPATIMUP, Porto, Portugal), the cervical cancer cell line SiHA, obtained from the American Type Culture Collection (Manassas, VA, USA), and primary human keratinocytes were included. The study was approved by the Institutional Review Board and was in accordance with medical and ethical guidelines in place in The Netherlands.

### Cell culture

Cells were maintained in standard culturing conditions. IPA220 and GP202 were cultured in RPMI (Life Technologies, Breda, The Netherlands) supplemented with 10% fetal calf serum, 100 U ml^−1^ penicillin, 100 *μ*g ml^−1^ streptomycin and 2 mmol l^−1^ L-glutamine (Life Technologies) (Gartner *et al*, 1996). The cervical cancer cell line SiHa was maintained in DMEM (Life Technologies) supplemented with 10% FCS, 100 U ml^−1^ penicillin, 100 *μ*g ml^−1^ streptomycin and 2 mmol l^−1^ L-glutamine (Life Technologies) (Steenbergen *et al*, 2004). Primary keratinocytes were cultured in serum-free keratinocyte growth medium (Life Technologies) supplemented with bovine pituitary extract (50 *μ*g ml^−1^), epidermal growth factor (5 ng ml^−1^), penicillin (100 U ml^−1^), streptomycin (100 *μ*g ml^−1^) and L-glutamine (2 mmol l^−1^) (Life Technologies) (Steenbergen *et al*, 1996).

### DNA and RNA isolation procedures

DNA of the primary gastric tumour tissues was isolated as described before using a commercially available DNA isolation kit (QIAamp microkit; Qiagen, Hilden, Germany) (Weiss *et al*, 1999; Buffart *et al*, 2007). Briefly, areas of at least 70% of tumour cells were marked on a 4 *μ*m haematoxylin and eosin stained tissue section. Tumour tissue was macro dissected from adjacent serial 10 *μ*m sections, using a needle. After deparaffinisation, an overnight incubation at 37°C with sodium-thiocyanate (1 M) and a proteinase K treatment, DNA was extracted.

DNA and RNA of the 17 snap-frozen gastric carcinoma tissue samples and gastric cancer cell lines, SiHa cervical cancer cell line and primary keratinocytes was isolated using TRIzol reagent (Invitrogen, Breda, The Netherlands) according to the instructions of the manufacturer, with some modifications. Details are described elsewhere (Weiss *et al*, 1999, 2003) (http://www.english.vumc.nl/afdelingen/microarrays/). All DNA and RNA concentrations and purities were measured on a Nanodrop ND-1000 spectrophotometer (Isogen, IJsselstein, The Netherlands).

### Bisulphite treatment and real-time methylation specific PCR

Of each DNA sample, 500 ng was used for bisulphite treatment using a commercially available DNA modification kit (EZ DNA Methylation Kit™; Zymo Research, Orange, CA, USA).

Real-time methylation specific PCR (Q-MSP) was performed using primer sets targeting two regions within the *MAL* promoter (i.e. from −680 to −573 bp (M1) and −92 to −7 bp (M2) relative to the first ATG). Both regions within the *MAL* promoter were selected within the CpG island of the *MAL* promoter. Amplicons were detected and quantified using Taqman probes. The housekeeping gene *β-actin* was chosen as a reference for total DNA input measurement.

Q-MSP reactions were carried out in a total reaction volume of 12 *μ*l containing 50 ng bisulphite treated genomic DNA, 417 nM of each primer, 208 nM probe and 2 × QuantiTect Probe PCR Kit master mix (Qiagen, Westburg, Leusden, The Netherlands). For both *MAL* M1 and M2 regions, the PCR reaction was performed for 45 cycles (15 s at 95°C and 60 s at 59°C) with an initial denaturation of 15 min at 95°C. For each Q-MSP a standard curve of serial dilutions of bisulphite-treated DNA (50, 5, 2.5, 0.5 and 0.25 ng) of the SiHa cervical cancer cell line was used. All samples were analysed in duplicate. As a negative control, multiple water samples, unmodified genomic DNA obtained from SiHa cells and unmethylated DNA obtained from primary keratinocytes were included. To determine the relative quantity of methylation, we calculated the ratios between *MAL* M1 and *MAL* M2 methylated DNA *vs β-actin* DNA (average quantity of methylated *MAL* DNA/average DNA quantity for *β-actin* × 1000).

### Real-time RT–PCR

Of each RNA sample, 1*μ*g was reverse transcribed to cDNA using oligo(dT)_20_ Primer (Invitrogen) with AMV reverse transcriptase (Promega, Leiden, The Netherlands). RT–PCR was performed in a total reaction volume of 25 *μ*l, containing 22.5 *μ*l master mix and 2.5 *μ*l cDNA (25 ng). The master mix contained 12.5 *μ*l of SYBR Green PCR master mix (Applied Biosystems, Nieuwerkerk a/d IJssel, The Netherlands) and 0.5 *μ*M of each primer. All samples were analysed in duplicate in a 7300 Real-time PCR System (Applied Biosystems). Amplification was performed in 50 cycles of 95°C for 15 s and an annealing temperature of 60°C for 1 min, with an initial denaturation step of 5 min at 95°C. Relative expression levels were determined from the obtained Ct values and the 2DDCt method, using *snRNP U1A* as reference (Livak and Schmittgen, 2001), and transformed into a ln scale. Primary keratinocytes and the SiHa cervical cancer cell line were used as positive and negative controls respectively. Primer sequences are described earlier (Wisman *et al*, 2006; de Wilde *et al*, 2008; Wilting *et al*, 2008).

### Statistical analysis

Receiver operator characteristic (ROC) curves were analysed for assessing the best cutoff value for methylation, on all FFPE samples, assuming that normal gastric mucosae are unmethylated and gastric carcinomas are methylated. Cutoff points were chosen based on the point on the ROC curve showing 100% specificity. Positivity for each methylated promoter region was considered when a specific sample had a ratio of M1/*β-actin* × 1000 or M2/*β-actin* × 1000 above the respective cutoff value. A sign test was used for testing significance of differences in frequencies of M1 *vs* M2 methylation.

Mann–Whitney U-test was used to determine significance of differences in expression values between methylated and unmethylated gastric carcinomas. Survival analysis was performed using the Kaplan–Meier method, using the survival length starting from the day of surgery of the primary tumour to the date of death due to gastric cancer (event) or to the last day of clinical follow-up (censored). Differences in survival length were analysed using the log-rank test. Multivariate analysis was performed using a Cox's proportional hazard regression model in a forward stepwise method for variable selection. Gender, histological type, tumour stage (T-stage) and lymph node stage (N-stage) were entered into the analysis. *χ*^2^ test was used for calculating differences in methylation status and tissue type, gender of the patient, histological type of the tumour and tumour stage. *t*-Test was used to evaluate age related differences in methylation status (SPSS 14.0 for Windows, Chicago, IL, USA). *P*-values below 0.05 were considered to be significant.

## Results

### Frequent *MAL* promoter methylation in gastric carcinomas

**Figure 1:**
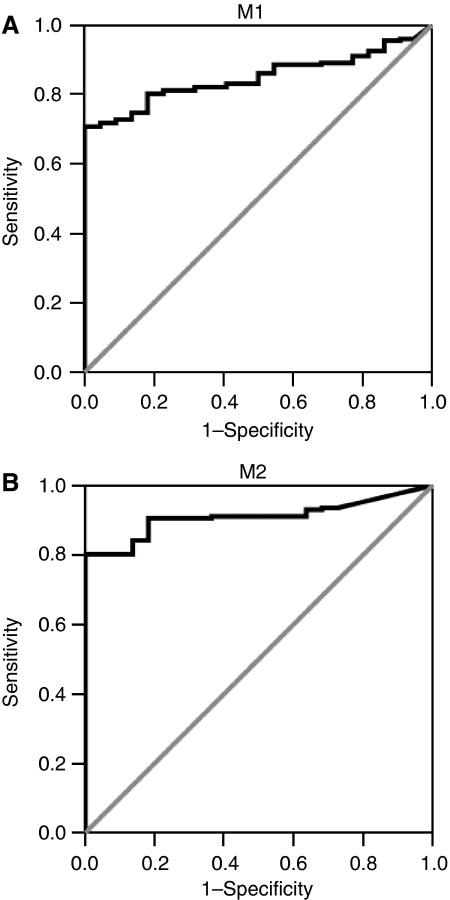
Receiver operator characteristics of (**A**) M1 promoter methylation and (**B**) M2 promoter methylation in 202 gastric cancers and 22 normal gastric mucosa samples (FFPE samples only), assuming that normal gastric mucosae are unmethylated and gastric carcinomas are methylated. Chosen cutoff levels for methylation were 95 and 22 for M1 and M2 promoter regions, respectively. This yielded a specificity of 100% for both promoter regions and a sensitivity of 71% for M1 promoter region and 80% for the M2 promoter region.

The chosen cutoff values of methylation for the M1 and M2 promoter regions, based on the ROC curve analysis, were ratios of relative methylated DNA quantities of M1/*β-actin* × 1000 and M2/*β-actin* × 1000 above 95 and 22, respectively, yielding a specificity of 100% for both promoter regions and a sensitivity of 71 and 80% for M1 and M2 promoter regions, respectively. ROC curves for both M1 and M2 promoter regions are shown in Figure 1.

Both gastric cancer cell lines, IPA220 and GP202, showed methylation of both M1 and M2 promoter regions. Of all 202 gastric adenocarcinomas tested, 143 carcinomas (70.8%) showed methylation of the M1 promoter region and 162 carcinomas (80.2%) showed methylation of the M2 promoter region. Methylation prevalences of M1 and M2 promoter regions were significantly different (*P*=0.004). Dense methylation, that is, methylation of both M1 and M2 promoter regions, was detected in 133 (65.8%) of the carcinomas. Thirty carcinomas (14.9%) were unmethylated. All normal gastric mucosa samples were unmethylated for both regions. *χ*^2^ test yielded a significant difference between methylation status of gastric carcinomas and normal gastric mucosa tissues (*P*<0.001). An overview of the methylation status for both promoter regions is given in Table 1.

### Correlation of *MAL* promoter methylation and survival

**Figure 2:**
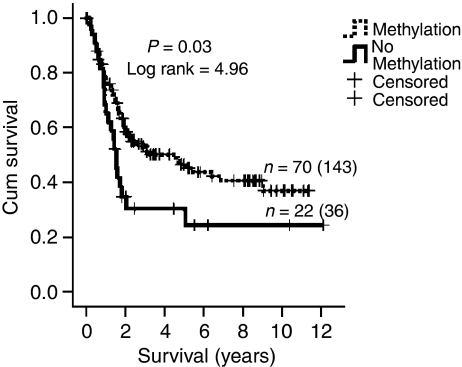
Kaplan–Meier survival analysis of 179 patients with primary gastric cancers assessed for the methylation status of the M2 region (−92 to −7 bp) within the *MAL* promoter. Patients with primary gastric cancers methylated for the M2 promoter region (*n*=143) showed a significantly better survival compared to patients (*n*=36) with gastric cancers without M2 promoter methylation (*P*=0.03; log rank=4.96). The number of patients who died of gastric cancer (events) is 70 and 22, respectively.

Follow-up data was available for 200 out of 202 patients. Only patients without distant metastasis at the time of surgery (M0) were included, leaving 179 patients for the survival analysis. Patients with a gastric carcinoma methylated for the M2 promoter region had a significantly better survival compared with patients with tumours unmethylated for the M2 promoter region (*P*=0.03) (Figure 2). No significant correlation was found between M2 promoter methylation and age or gender of the patient, histological type of the tumour, T-stage and N-stage (Table 2). In addition, no significant correlation between M1 promoter methylation status and clinicopathological characteristics, including survival, was found.

Multivariate analysis revealed only N-stage, T-stage and *MAL* M2 promoter methylation status, in order of significance, to have independent prognostic value (Table 2).

### *MAL* promoter methylation is associated with reduced gene expression

**Figure 3:**
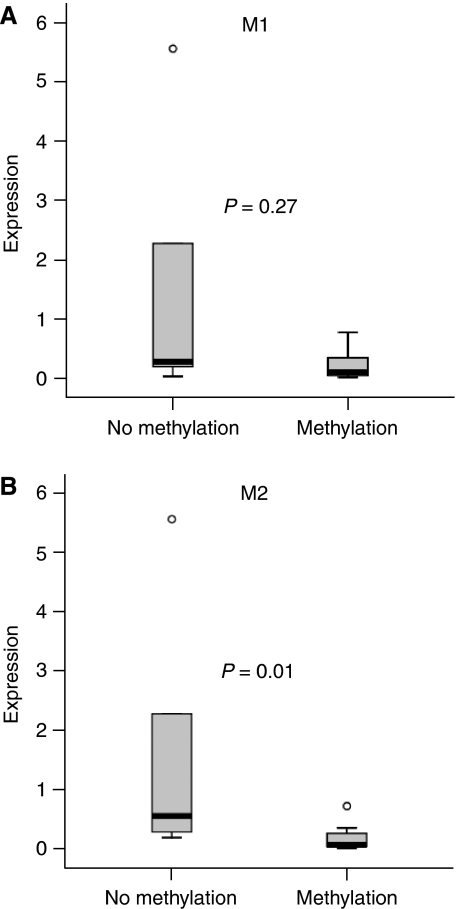
Box plots of the relative expression values of 17 gastric carcinoma tissue samples methylated and unmethylated for the M1 (**A**) and M2 (**B**) promoter regions. Gastric carcinomas methylated for the M2 *MAL* promoter region show significantly lower expression of the *MAL* gene compared with unmethylated gastric carcinomas (*P*=0.01).

Reduced expression of *MAL* relative to the housekeeping gene *snRNP U1A* was observed in both gastric cancer cell lines compared with primary keratinocytes that did not show *MAL* promoter hypermethylation. Of the 17 gastric cancer tissue samples tested for *MAL* mRNA expression, 11 (64.7%) were methylated for M1, 11 (64.7%) for M2 and 9 (52.9%) samples showed methylation of both regions. Gastric carcinomas with M2 promoter methylation showed significantly lower expression of the *MAL* gene compared with M2 unmethylated gastric cancers (*P*=0.01) (Figure 3, Table 3). For the M1 region, no significant differences in *MAL* mRNA expression were found between methylated and unmethylated tumours.

## Discussion

Gastric cancer is a common disease with generally a poor prognosis (Parkin *et al*, 2005). Biomarkers can be used to predict prognosis and optimise therapeutic strategies. Hypermethylation of the *MAL* promoter has been shown in colorectal and oesophageal cancers and *MAL* has been proposed as a putative tumour suppressor in these cancer types (Mimori *et al*, 2003; Kazemi-Noureini *et al*, 2004; Mori *et al*, 2006). Moreover, it has been proposed as a candidate marker for early detection of these cancers, as methylation of *MAL* could already be detected in precursor lesions (Lind *et al*, 2007, 2008; Mimori *et al*, 2007). In this study we show that *MAL* might have a similar role in gastric cancers, as methylation of *MAL* is detected at high frequency in gastric cancers and not in normal gastric mucosa samples.

In this study, two regions within the *MAL* promoter, M1 and M2, were analysed. Q-MSP analysis showed methylation of both promoter regions in the two gastric cancer cell lines analysed, indicating that methylation actually occurs in gastric epithelial cells. Earlier studies showed protein expression of MAL by immunohistochemistry in normal gastric mucosa. Strong expression of MAL was observed in parietal and chief cells, but not in muscle and submucosa cells (Marazuela *et al*, 2003). Gel-based MSP analysis revealed a small subpopulation of unmethylated cells for both the M1 and M2 promoter regions in these cell lines (data not shown). Expression of *MAL* was hardly detected in both cell lines indicating that methylation is probably the main mechanism of downregulation of this gene. In gastric cancers, methylation of the M2 region was more frequently observed compared with the M1 region (80.2 *vs* 70.8%). In consistence with what has been described by Lind *et al* (Lind *et al*, 2008), an unequal distribution of DNA methylation within the *MAL* promoter was observed in gastric cancers, with the highest frequencies of methylation in the region closest to the transcription start site. M2 region is located around the transcription start site.

Results of this study show that *MAL* may serve as a prognostic marker in gastric cancer, as patients with tumours methylated for the M2 region show significantly better survival compared with patients with tumours unmethylated for *MAL* or methylated only for the M1 region. This survival benefit was independent of other clinicopathological data such as age, gender, histological type of the tumour, tumour stage and lymph node status. Interestingly, also in Hodgkin's lymphoma patients a similar association was found with a significantly worse survival in patients whose tumours expressed the MAL protein compared with patients with tumours lacking MAL expression (Hsi *et al*, 2006), indicating a prognostic value of *MAL* also in other cancer models.

The finding that inactivation of *MAL* by methylation gives a better prognosis for the patients may seem contradicting with a putative tumour suppressor function of this gene in gastric cancer. However, this finding has been observed earlier for the mismatch repair gene *hMLH1*, which also has a tumour suppressor function. Inactivation of this gene leads to microsatellite instability of the tumour and patients with microsatellite instable tumours have a better prognosis compared with patients with microsatellite stable tumours (Ribic *et al*, 2003; Parc *et al*, 2004; Beghelli *et al*, 2006). Therefore, tumours without *MAL* methylation might have a different biology overall, which could relate to poorer clinical outcome, rather than the outcome being dependent on *MAL* itself. However, this study was performed retrospectively on archival material, and therefore, rather should be considered as hypothesis generating. Actual clinical implementation of *MAL* promoter hypermethylation as a diagnostic marker requires further validation in a prospective study.

To test the biological relevance of *MAL* promoter hypermethylation, in a subset of gastric cancers the association between *MAL* promoter hypermethylation and mRNA expression was analysed. Results showed lower expression of *MAL* in gastric cancers methylated for the M1 or M2 region. The association between promoter methylation and reduced mRNA expression strengthens the biological relevance of *MAL* methylation and supports a putative role as tumour-suppressor gene. However, correlation between reduced expression and methylation of *MAL* was only significant for the M2 region, indicating that M2 methylation would have more biological relevance. Two out of 17 gastric cancers showed reduced *MAL* mRNA expression whereas the gene was unmethylated for both promoter regions. This indicates that other regulatory mechanisms of *MAL* silencing also exist in gastric cancer, which may include DNA copy number loss, other epigenetic mechanisms, altered expression of transcription factors regulating *MAL* or microRNAs targeting the *MAL* gene.

In summary, this study shows frequent promoter hypermethylation of *MAL* in gastric cancers, and not in normal gastric mucosa samples. Promoter hypermethylation of *MAL* is associated with downregulation of its expression, especially when methylation occurs at the M2 region within the promoter. Methylation of this region within the *MAL* promoter correlates with a significantly better survival of the patients. Altogether, these results pinpoint *MAL* as a putative tumour-suppressor gene with a role in gastric cancer, which may serve as an independent prognostic marker for clinical outcome of gastric cancer patients.

## Figures and Tables

**Table 1:** Overview of methylation status of M1 (−680 to −573 bp) and M2 (−92 to −7 bp) regions within the *MAL* promoter for 202 gastric carcinoma tissues and 22 normal gastric mucosa tissues

	**Carcinomas *n*=202**	**Normal mucosa *n*=22**	***P*-value**
No methylation	30 (14.9%)	22 (100%)	<0.001
M1 methylation	143 (70.8%)	0 (0%)	<0.001
M2 methylation	162 (80.2%)	0 (0%)	<0.001
dense methylation	133 (65.8%)	0 (0%)	<0.001

*χ*^2^ test yielded a significant difference between methylation status of gastric carcinomas and normal gastric mucosa tissues (*P*<0.001).

**Table 2:** Overview of patient and tumour characteristics of the 179 tumours used in the univariate and multivariate survival analysis

	**Univariate analysis**		
	**Methylated**	**Unmethylated**	***P*-value**
Age (years)	72 (52–96)	71 (54–87)	NS
			
*Gender*			
Male	88 (62%)	20 (56%)	NS
Female	55 (38%)	16 (44%)	
			
*Histological type*			
Intestinal	100 (70%)	23 (64%)	NS
Diffuse	18 (13%)	7 (19%)	
Mixed	25 (17%)	6 (17%)	
			
*T-stage*			
T1	10 (7%)	2 (6%)	NS
T2	50 (35%)	16 (44%)	
T3	78 (55%)	18 (50%)	
T4	5 (3%)	—	
			
*N-stage*			
N0	41 (29%)	9 (25%)	NS
N1	62 (43%)	15 (42%)	
N2	32 (22%)	8 (22%)	
N3	7 (5%)	4 (11%)	
Unknown	1 (1%)	—	
			

	**Multivariate analysis**		
	**HR**	**95% CI**	***P*-value**
Gender			0.94
Histological type			0.48
T-stage	1.73	1.23–2.42	0.001
N-stage	1.52	1.20–1.91	0.001
*MAL* M2 methylation	0.59	0.36–0.96	0.03

CI=confidence interval; HR=hazard ratio.

Absolute number and percentages are given for gender, histological type, tumour stage (T-stage) and lymph node status (N-stage). Age is given as mean age and range. None of the clinicopathological characteristics were significantly correlated with M2 promoter methylation status (*P*=NS). Multivariate analysis showed that T-stage, N-stage and M2 methylation status are prognostic variables for patient outcome.

**Table 3:** Relative ln transformed expression values (E) and methylation status for the M1 and M2 regions within the *MAL* promoter of the two gastric cancer cell lines and 17 gastric carcinoma tissues

**Sample**	**E**	**M1**	**M2**
IPA220	0.003	Methylated	Methylated
GP202	0.0003	Methylated	Methylated
1	0.35	Methylated	Methylated
2	0.24	Methylated	Methylated
3	0.06	Methylated	Methylated
4	0.72	Methylated	Methylated
5	0.10	Methylated	Methylated
6	0.05	Methylated	Methylated
7	0.77	Methylated	Unmethylated
8	0.28	Unmethylated	Unmethylated
9	0.19	Unmethylated	Unmethylated
10	2.28	Unmethylated	Unmethylated
11	0.28	Unmethylated	Methylated
12	0.03	Unmethylated	Methylated
13	0.03	Methylated	Methylated
14	0.04	Methylated	Methylated
15	5.56	Unmethylated	Unmethylated
16	0.01	Methylated	Methylated
17	0.33	Methylated	Unmethylated
